# Obstetric Auscultation

**Published:** 1839-10

**Authors:** 


### Art. III.

*Die geburtshülfliche Auscultation.* Von Dr. Herm. Franz Naegele. —*Mainz*, 1838. 8vo, pp. 140.

*Obstetric Auscultation.* By Herman Francis Naegele, m.d. — *Mayence*, 1838.

Although we have, on more than one occasion, discussed the merits of obstretric auscultation, particularly in reviewing the works of M. Hohl, (vol. I. p. 85,) still we have not had until now an opportunity of entering exclusively upon the consideration of this important subject; having, however, recently obtained the little work above mentioned, we lose no time in earnestly requesting the attention of our readers to it. The author is the son of a distinguished professor of that name at Heidelberg, who has long been honorably known as one of the first obstetric teachers upon the continent. Although a very young man, Dr. N. has already distinguished himself as the author of some interesting papers in the German journals, one of which we have ourselves recently noticed; and he has been for some years closely occupied in collecting materials for the present undertaking. The work itself is purely practical; and in his preface the author refers his readers to the elaborate work of Professor Hohl, for the historical part of obstetric auscultation.

We need scarcely observe that Dr. Naegele prefers the stethoscope to using the ear alone; the instrument which he uses (as far as we can judge from his description of it) does not appear to differ materially from those commonly used in this country, except that it is somewhat thicker than usual; in giving his directions how to apply and use it, he strongly deprecates (with Laennec) stooping with the head downwards over the patient, as such a position must necessarily produce much congestion of the head, and so much buzzing in the ears as materially to interfere in the diagnosis of those sounds which are faint and but indistinctly heard. For the same reason he advises the operator not to auscult for many minutes at a time, without removing his ear from the disc of the instrument; for otherwise the pressure will more or less obstruct the free circulation of blood in the external ear, and thus produce souffle-like sounds, which may tend to mislead him.

In describing the situation of the uterine souffle, Dr. N. observes that it may always be heard in one or both inguinal regions.

"From this part it usually extends, although mostly on one side, towards the hypochondrium, or more forwards towards the umbilicus; and the limits within which it can be heard cannot be marked very exactly. In the majority of cases they at any rate correspond to the circumference of the placenta; in some it extends from both groins over the whole uterus; in others it is more confined to the lower portions—there is no part of the uterus which can be reached by the stethoscope in which we have not heard it." (p. 17.)

Dr. Naegele considers that the uterine souffle may be detected for the first time as early as the beginning of the fourth month. He distinguished it in 20 cases out of 35 at the fifteenth week; and in three cases only at the fourteenth week. This agrees with professor Hohl's observation; but we have already stated that Dr. Evory Kennedy, the present master of the Dublin Lying-in Hospital, has detected it at the twelfth, eleventh, and perhaps even tenth week of pregnancy—a fact of which Dr. N. does not seem aware. "In the early periods of pregnancy the uterine sound is more feeble; it extends over the whole uterus, and is heard most distinctly immediately over the symphisis pubis." (p. 18.) He warns the beginner not to press the stethoscope deeply into the abdominal integuments, as this will only hurt the patient and not assist him in his diagnosis.

The author differs from Professor Hohl, respecting the changes of tone which the uterine sound undergoes, upon the accession of a labour pain; we must confess that the description which Professor H. has given of them (see *Br. and For. Med. Rev.* vol. I. p. 93,) accords with our own experience; it is not very easy to make an accurate auscultation during a pain, for reasons which are self-evident; still, however, the peculiar piping character of the uterine sound at these moments cannot be mistaken, and may be heard best in the inguinal region; the difficulty of ausculting the fundus during a pain is perhaps the reason why we were not aware of the interesting fact which Dr. N. has mentioned, viz. that at the moment of greatest contraction the uterine sound generally ceases entirely over the fundus and body of the organ, although, as he also observes, it continues audible in the groins.

That the uterine sound generally ceases at the moment of the placenta being detached from the uterus is a fact which has been long since pointed out by Dr. Kennedy, in his excellent little work on this subject; we regret that the auscultation of the uterus during the first forty-eight hours after labour has not been made the subject of enquiry by our author, as we feel sure he would have noticed the souffle which has been described by Dr. Kennedy and confirmed by ourselves. (*Ib.*, vol. I. p. 91.)

The uterus does not long maintain that state of firm contraction in which it is usually found immediately after labour, and which must be looked upon as the reason why the souffle ceases to be heard at this moment, as also in the upper portions of the organ during the height of a pain; in the course of twenty minutes or half an hour the contraction gradually abates in power, the uterus increases in size, its vessels are again filled, and this state usually continues until about forty-eight hours have elapsed, when the uterus again begins to contract and to diminish in size, until it is not much larger than it was in the unimpregnated state. It is during this condition of slight relaxation that the lochia make their appearance; and, as far as we have the opportunity of observing, the uterine souffle after labour is chiefly confined to this period.

It is well known that the bruit which is heard in certain conditions of the heart and large vessels occasionally bears a strong resemblance to the uterine sound or souffle; this is most remarkably the case, as Dr. N. observes, in aneurismal varix, where a direct communication for the blood exists from an artery into a vein. "Dubois compares the uterine parietes, as respects their structure, with a tissue which consists of a natural varicose aneurism. Hohl fully agrees with him on this point, and carries it even further, since he considers the existence of the placenta uterina fully proved, and that the passage of the arteries into veins at this part is not effected by means of anastomosis, but rather by large cells." (p. 25.) We cannot imagine it possible for any one with an impartial mind to read and repeat the admirable observations of Hunter on this subject without coming to a similar conclusion. We consider that the facts connected with the placental circulation are as distinctly and satisfactorily established as any point in anatomy or physiology can be; we cannot, therefore, agree with our author, when he says that the vascular connexion between the uterus and placenta is far from being determined. "The souffle which is so uniformly heard in the inferior portions of the uterus arises, in our opinion, from the uterine arteries before they enter the uterus; and we observe that these vessels, as soon as they reach the broad ligament, assume a different character, become thicker than where they branched off from the main trunk, and, after having made numerous windings, sink into the substance of the uterus." (p. 28.) Dr. N. considers that the tortuosity of these vessels, their dilated state, and the thinness of their parietes, sufficiently account for the uterine souffle; we know, however, that these vessels are not so remarkable for their tortuous course in a first pregnancy as afterwards; and therefore must take this opinion with some degree of caution.

In remarking the variable character of the fœtal pulse and the sudden alterations to which it is subject, the author has very properly examined the rate, &c. of the heart shortly after birth, and finds that it presents the same sudden changes as in the heart of the fœtus before birth. "How variable is the pulse of a child, even in the first years of its life: to-day it is 80—to-morrow, without any assignable reason, we find it up to 160 or 180. The pulse varies with extraordinary rapidity, not only whilst it is awake, but in apparently placid sleep—how rapidly it rises when the child moves, takes nourishment, or is subject to any mental excitement! we see it suddenly to rise 30, 40, or even 60 beats in a minute, changes which must tend greatly to confuse the practitioner who is not familiar with them, and show why the most eminent physicians place so little value on the pulse in diseases of children." (p. 37.)

We have already shown, in a quotation from Professor Kilian's work, (*Rev.*, vol. I. p. 90,) that nothing which accelerates the pulse of the mother affects that of the child; the author of the treatise before us has repeatedly examined females immediately after violent exercise, when the action of the mother's heart was greatly increased, and beating at the rate of 90, 100, or even 120 strokes in a minute, and yet the action of the fœtal heart was neither increased in rapidity or power. In cases, also, where this effect had been produced by drinking ardent spirits, the fœtal heart remained unaffected. In cases of slight catarrhal fever, and even of severe pleuritis and pneumonia, the fœtal heart was found beating at the normal rate: these and other equally well-marked cases, especially where the dyspnœa was very severe, show that such circumstances do not affect the fœtal heart, as supposed by Professor Hohl. One of the author's cases is of considerable interest, where, even in a state of really dangerous syncope, the fœtal heart was heard beating with its usual strength and rapidity; he has observed the same fact in cases of hemorrhage.

The earliest period at which Dr. Naegele has detected the sound of the fœtal heart has been the eighteenth week of pregnancy: the cases in which it was heard thus early were primiparæ. "Before the first half of pregnancy had elapsed we have been enabled to recognize the sound of the heart in 30 cases out of 50. Although our attempts were frequently unsuccessful before the twentieth week, yet when the second half of pregnancy had commenced, we usually succeeded; now and then the sound of the fœtal heart did not become audible until the sixth month." (p. 48.) This agrees pretty much with the observations of Dr. Kennedy, who says, "although we have in a few cases detected this sound, even before the expiration of the fourth month, it will not in the majority be possible until a later period; and in those cases where it can be detected about this time, it is sometimes so delicate and feeble as to render it necessary for the individual exploring to have an ear well trained to stethoscopic sounds." (p. 101.) In fact, the discrepancy as to the earliest moment of its becoming audible is scarcely appreciable, if we bear in mind that Dr. K. has reckoned by calendar months, whereas the German authors do it either by weeks or lunar months, and hence consider that the duration of pregnancy is ten months, or forty weeks.

In examining the sound of the fœtal heart during a labour pain, Dr. Naegele has found it to become indistinct or quite inaudible, in proportion to the strength and energy of the contraction. He considers that this results from the sound which is produced by the uterine fibres and abdominal muscles, when contracting. It is well known that a peculiar grinding sound, if we may thus describe it, is heard when the stethoscope is placed upon a muscle in action; and Dr. N. compares the sound which now overpowers that of the fœtal heart to that which is produced by the masseter muscle during mastication; this may possibly be the case, but we confess that the difficulty of keeping the patient either still or quiet at these moments is such as to be quite sufficient to render such slight sounds inaudible.

The author has very properly noticed the fact of the sound of the fœtal heart becoming more distinct when the membranes have ruptured, and a portion of the liquor amnii come away; but we find no mention of a fact which attends this change, and which ought not to be omitted, viz. that now the sound, although more distinct, is heard over a smaller extent; whereas, when there is a stratum of liquor amnii between the thorax of the fœtus and the uterus, the sound, although less clear, is diffused over a greater extent. "If in cases where the head presents, the fœtal heart has been heard in one of the superior abdominal regions, previous to the discharge of the liquor amnii, it will continue to descend during the latter part of labour, so that in proportion as it becomes inaudible in the superior abdominal region, it becomes distinct in the inferior one. At this latter part the heart may be heard distinctly until the head has completely entered the vagina: the moment this is born all traces of the fœtal heart above the pubes cease."

In describing the sound produced by the circulation in the umbilical cord, we regret that the author has made no mention of Dr. Evory Kennedy's highly interesting observations on this subject. These were published as early as 1833, and contain much original matter. Dr. K. has also remarked the peculiar souffle of the umbilical cord, and rightly ascribes it to pressure, having succeeded in producing it at pleasure. This souffle of the cord is chiefly heard when a portion of it is pressed between a limb or the back of the child; it does not require to be coiled or twisted round any part to produce this effect, as Dr. N. has rightly observed; but this was shown long ago by Dr. Kennedy, who succeeded in *feeling* the cord, as it passed across the child beneath the thin uterine and abdominal parietes of the mother; he states it as being easily distinguished, prominent, rolling under the finger, and pulsating." (p. 124.) In the same case to which we have now alluded, Dr. K. made an experiment, which is too interesting to pass unnoticed. "Having fixed the funis against the limb of the child, between the finger and thumb of the left hand, I made a gentle pressure with the fore-finger of the right hand on the cord, keeping my ear applied to the stethoscope, the other end of which was over the funis, at a point nearer its insertion into the placenta. The pulsation which, up to the moment of my making this pressure, was remarkably strong and distinct, became converted into a souffle; and on increasing the pressure it immediately ceased, recommencing the moment I discontinued it. I then removed the stethoscope to the spot where I had discovered the heart's pulsation, and repeated the experiment as above. The action of the heart at first became laboured, but fuller; afterwards it became fluttering and indistinct; and not judging it safe to continue the pressure any longer, lest the child should suffer, I removed it, when the action became regular as before, but somewhat quicker." (p. 125.) We cannot help thinking that the facts related in the above quotation prove the existence and nature of the funic souffle far more distinctly than the various reasons which Dr. Naegele has given; at the same time we must in justice say that the care with which he has made these observations reflects much credit upon him; and, although we have no doubt whatever of their being original as far as he is concerned, still, nevertheless, the merit of priority must rest with Dr. Kennedy.

In speaking of the stethoscope, as connected with the diagnosis of pregnancy, Dr. Naegele mentions a sound which, as far as we know, has never been made available for this purpose: we allude to that produced by the movements of the fœtus. "Among these signs (of pregnancy) we must enumerate the sound produced by the plunging movements of the child's limbs, which we are frequently able to hear much earlier than they can be felt by the hand of the practitioner, when applied upon the patient's abdomen, or indeed by the patient herself. Although we are inclined to look upon the audible movement of the child's limbs as one of the earliest certain signs of pregnancy, this symptom does not present itself in every case or at the same period." (p. 62.) The actual percussions which are occasionally transmitted to the ear when auscult-ing the gravid uterus during the last weeks of pregnancy, must be familiar to every one; but we are not aware of their having been noticed at an early period, as described by Dr. Naegele, who has, to the best of our knowledge, the merit of having first called the attention of the profession to this fact.

Much as our author respects the stethoscope, as a valuable addition to our means of diagnosis, still, however, he is very far from looking upon it as a substitute for manual exploration.

"We are thoroughly convinced that this latter mode of examination will never be rendered unnecessary, and that auscultation will never succeed in taking the place of examination per tactum. Auscultation is an important addition to the common method of examining, and the combination of these two means of diagnosis enables us to draw much more distinct conclusions from their mutual results; in illustration of this, let us suppose the following cases: for instance, we have reason to suspect pregnancy, but we can hear no sound of the fœtal heart, the fœtus may be dead; if we hear the uterine sound in this case, and other symptoms speak in favour of pregnancy (such as feeling a portion of the child presenting), we are led to the conclusion that the patient is pregnant but that the child is dead. If, on the other hand, in a case of doubtful pregnancy, we hear no sound whatever, after the most careful auscultation, and cannot detect any of the usual symptoms to be found upon manual exploration, we shall be enabled to decide, with so much more certainty, that our patient is not pregnant. In short each of these modes of examination offers its peculiar advantages, the greatest, however, are when they are combined, where they mutually act as checks upon each other." (p. 64.)

With regard to the diagnosis of pregnancy where there are twins, Dr. Naegele considers that the assertion of the uterine sound being much louder and more extended, especially in two different portions of the uterus, from the presence of two placenta, is not borne out by experience. He is inclined to think that the presence of twins in the uterus can only be ascertained by hearing the sound of the two fœtal hearts. "Whatever may be the position of the children, the pulsations will never be heard at the same horizontal line of the mother's abdomen. This difference, with regard to the spot at which the pulsations are heard most distinctly, is well worthy of notice, because the pulsations of the two fœtal hearts are frequently perfectly synchronous." (p. 67.) In a case of twins which the author ausculted, he observes, "we perceived distinctly the pulsation of a fœtal heart in the left inferior abdominal region, showing that the back of a fœtus was in this direction; in the right inferior abdominal region we could hear nothing, whereas in the right upper abdominal region (near the hypochondrium), the pulsation of another heart was audible, the distinctness of which plainly showed that the organ itself was at no great distance: in the left upper abdominal region, on the other hand, we could detect nothing of the sort, both hearts corresponded exactly with each other in point of rhythm." (p. 68.) This isochronism of the two fœtal hearts is by no means always the case; generally speaking, there is a considerable difference between them, both in rhythm and strength of pulsation. These points cannot be ascertained by one person, the two fœtal pulses must be ausculted at the same moment by two observers, and thus, however small the difference may be between them, it will be correctly determined.

With respect to the sounds which are heard in cases of extra-uterine pregnancy, Dr. N. has not been fortunate enough to meet with a case of this rare description; in a case of ventral pregnancy which we have had the opportunity of ausculting, and where the child was distinctly placed across the lower part of the abdomen, the bruit over the whole lower and anterior half was so extravagantly loud (if we might so express ourselves), as instantly to strike even the most inexperienced in the art of auscultation; we ourselves had not the opportunity of hearing the fœtal pulse, the child having evidently died about twenty-four hours before we saw the patient for the first time; the sound, however, had been recognized by a gentleman who had ausculted the abdomen, and, as far as we could ascertain, in nowise differed from that of the fœtal heart under ordinary circumstances. From this period the loud souffle became day after day more circumscribed, until when we last ausculted her (an interval of four or five weeks), it was confined to a small space and was very feeble. In speaking of the various species of deception to which auscultation of the abdomen may give rise, Dr. Naegele has observed that in some cases the sound of the mother's heart may be heard over a remarkable extent, even as far as the ossa ilia. He attributes this "to its being transmitted along the intestinal tube when filled with gas; the same effect may be produced where the abdomen has been distended by some morbid growth, and where other deceptive symptoms, as cessation of the catamenia, swelling of the breasts, &c. may lead to the supposition of pregnancy; if we apply the stethoscope in such a case, we hear a pulsation, and if the circulation of the patient be quicker than usual, it is considered to proceed from a fœtus in utero, and thus the case is pronounced to be pregnancy." (p. 71.) He has quoted a case, related by Dubois, so remarkable, that we cannot help giving a brief account of it.

"A young woman, in whom the menses had ceased for five months and a half, applied for admission at the Maternité under the supposition that she was pregnant; the size of the abdomen corresponded with the date of the cessation of her menses, and she assured him that she felt the child move. In about a month after, Dubois ausculted her, and found a double pulsation, varying from 128 to 130 strokes, at the lower part of the abdomen on the left side. Happening shortly afterwards to feel her pulse, he was astonished to find it beating at precisely the same rate. On repeating his auscultation he discovered that these double pulsations became more and more distinct as he approached the epigastrium, so that it was impossible not to recognize their real source; the sound of the patient's heart extended over the whole abdomen, and at its lower portion was so feeble as to be easily mistaken for the sound of a fœtal heart; upon making a careful examination, per vaginam, it proved that she was not pregnant." (pp. 72-3.)

In determining the situation of the placenta, Dr. Naegele considers that the souffle, which is commonly heard in both groins, is more distinct and over a larger space on that side at which it is attached; in ten cases of adherent placenta, the introduction of the hand has confirmed this observation. The industry and zeal which the author has displayed in determining these interesting points, are not less creditable to him than the talent by which they have been directed. The result of his observations shows a curious discrepancy from what had hitherto been considered as an established fact, respecting which side of the uterus the placenta is most frequently attached to, viz., that it is the left and not the right side, as commonly supposed. In 600 cases which he carefully ausculted, "the placenta was found in 238 cases on the left side and in 141 cases on the right side; of the remaining ones, no uterine sound was heard at all in twenty cases, and in 160 others it was too weak or too much limited to the inguinal regions to give any result; in seven only was the placenta distinctly in the fundus uteri." (p. 85.)

The author has noticed a peculiar piping, hissing character in the uterine sound in cases where the placenta was situated in the vicinity of the os uteri; he also confirms the observation of M. Hohl, viz., that this sound is met with where there is much of those calcareous depositions which are occasionally found in the placenta.

In determining whether the child be alive or not, Dr. N. considers that the stethoscope affords us the only *certain* means of forming a correct diagnosis.

"On detecting the sound of the fœtal heart, we can unhesitatingly decide that the child is alive, and we coincide most strongly with the assertion of Professor Hohl, in declaring ourselves opposed to the opinion of those authors who will not allow our not hearing the fœtal heart to be a proof of the child's death. If the practised auscultator be unable to detect any trace of the fœtal heart at a period of pregnancy, when it is wont to be heard with distinctness, not even by repeated careful examinations of the patient in different positions, provided the presence of pregnancy be satisfactorily established, there can be no doubt but that the fœtus is dead." (p. 91.)

We cannot altogether agree with Dr. Naegele in denying the correctness of Professor Hohl's remark, that the uterine souffle ceases when the fœtus dies; this sound continues for some time after the child's death in utero, and betrays little or no perceptible change in its strength or clearness; as, however, expulsion is seldom retarded beyond a few weeks after this occurrence, we have not often had an opportunity of determining the fact. The author is certainly right if Professor Hohl's observation refers to the auscultation of the uterus immediately after the child's death; but we have strong reason to doubt whether the uterine souffle will retain its full degree when three or four weeks have elapsed; indeed, his own observations appear to confirm this opinion, for in the case to which he refers in illustration (p. 95), he says, "the uterine sound was heard loudly in both inguinal regions, and extended from thence, *although with less power*, over the whole abdomen."

Dr. N.'s observations on the effects which prolonged uterine action will produce on the circulation of the child, although doubtless well known to experienced practitioners, are not the less worthy of notice.

"The life of the fœtus is greatly endangered when the progress of the labour, after the escape of the liquor amnii, is unnaturally protracted, and the uterus eventually remains in a hard, contracted state, whether this arises from faulty position of the child, or want of proportion between it and the passages; the closer the uterus contracts upon the child's body, the more is its circulation obstructed, and at length stopped altogether." (p. 99.) . . . In a considerable number of cases where the pains have been remarkably long and powerful, and where, after the escape of liquor amnii, one contraction of the uterus followed another so quickly, that the patient had scarcely time to get her breath, we have observed the pulse of the fœtal heart become slower and weaker; its rapidity has diminished from thirty to forty and even more beats, and yet the child has been born alive and perfectly strong and healthy; in such cases it was only able to resist the violence of the uterus during the short period that the labour lasted." (p. 102.)

In illustration of this interesting point we will merely quote a passage from the case which follows:

"The head descended in the first position into the pelvic cavity; during this storm of uterine contractions, the stethoscope was constantly applied, and at every brief interval of quiet, the fœtal heart was counted and gave the following result: the pulsation became every minute slower and in the following ratio, 140, 134, 136, 128, 130, 122, 120, 116, 100, 108, 100, 94, 86, 80, and, as the head was passing over the perineum, 75. It was now expected that an extremely feeble child would be born which could not live; but the result showed otherwise: when the contractions had continued for nearly three quarters of an hour, the child was expelled with a single pain; the funis pulsated very slow and feebly; but as soon as the child had made some efforts to breathe, the heart began to beat with strength, and shortly afterwards the child cried stoutly: the pulse was now 120 in the minute; in an hour after, during sleep, it was 110; the next day, during sleep, it was 126; and when the child was awake, 136." (p. 103.)

The author has given some interesting observations on the effects which partial detachment of the placenta, before labour, have on the uterine souffle; this gradually diminishes, and at length ceases entirely; at the same time the fœtal heart beats more slowly, and even sinks below the mother's pulse in point of frequency, it then makes several violent struggles and the child dies. If the head be pushed somewhat to one side, or slightly raised by the finger, a small quantity of dark blood will escape. For further particulars on this interesting subject, as also for two very instructive cases of prolapsus of the cord, we must refer our readers to the work itself. Dr. N. has called our attention to a remark made by Levret, respecting the situation of the placenta, viz., that the nearer this mass is attached to the os uteri, the nearer is the insertion of the cord to the lower edge of it. Dr. N., from having observed these circumstances in the above-mentioned cases of prolapsed funis, rather prematurely in our mind, deduces the following conclusion, viz., "the attachment of the placenta, in the vicinity of the os uteri, is undoubtedly one of the predisposing causes of prolapsus of the funis during labour; and this circumstance is the more to be dreaded, the nearer the insertion of the cord is to the lower edge of the placenta." (p. 114.) We must differ here most strongly from him. In all the cases of partial situation of the placenta over the os uteri, which we have had the opportunity of seeing (and they have been pretty numerous), especially where *merely* the edge of the placenta covered that of the os uteri, we have never met with prolapsus of the cord, nor do we recollect to have noticed the marginal insertion of it into the placenta, as above mentioned, in such cases; nor can we call to mind a single case of prolapsed cord which we could attribute to this cause, but rather to unusual quantity of liquor amnii, to want of regular contraction in the lower segment of the uterus, to death of the fœtus, &c. Dr. N. has tried Michaelis's method of replacing the funis without success, and finds that using the hand alone is preferable. We have already noticed Dr. Michaelis's observations in our Second Number, page 588, also in Number VI. page 523, to which we must refer our readers.

We thus close rather a long review of a short book; and this very circumstance is, we trust, of itself a sufficient proof how favorably we think of it. It contains a great deal of valuable and original matter in a small space; the style is simple, clear, and unaffected; and we earnestly recommend it to the perusal of our readers, the more so, because it contains many highly interesting observations which want of space precludes us from noticing. The cases which Dr. Naegele has given in illustration are peculiarly interesting, and will be found very instructive. The work is well worthy of translation.

